# Polaritonic Chemistry: Hindering and Easing Ground State Polyenic Isomerization via Breakdown of σ–π Separation

**DOI:** 10.1021/acs.jpclett.3c02081

**Published:** 2023-10-05

**Authors:** Marco Severi, Francesco Zerbetto

**Affiliations:** † Department of Chemistry G. Ciamician, University of Bologna, Via F. Selmi 2, 40126 Bologna, Italy

## Abstract

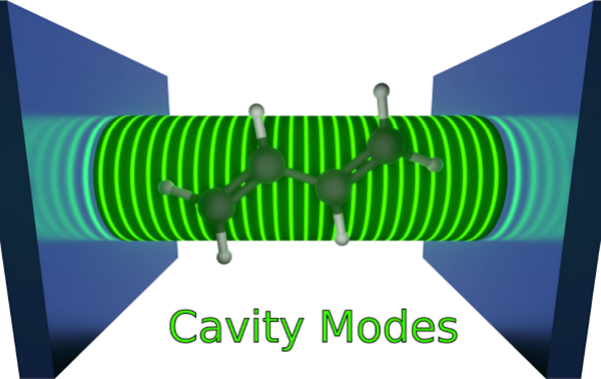

The ground state conformational isomerization in polyenes is a symmetry allowed process. Its low energy barrier is governed by electron density transfer from the formal single bond that is rotated to the nearby formal double bonds. Along the reaction pathway, the transition state is therefore destabilized. The rules of polaritonic chemistry, i.e., chemistry in a nanocavity with reflecting windows, are barely beginning to be laid out. The standing electric field of the nanocavity couples strongly with the molecular wave function and modifies the potential energy curve in unexpected ways. A quantum electrodynamics approach, applied to the torsional degree of freedom of the central bond of butadiene, shows that formation of the polariton mixes the σ–π frameworks thereby stabilizing/destabilizing the planar, reactant-like conformations. The values of the fundamental mode of the cavity field used in the absence of the cavity do not trigger this mechanism.

Molecular and polymeric systems with a backbone of π-conjugated carbon atoms are of fundamental interest in chemical reactivity where their behavior is rationalized in terms of symmetry, frontier orbitals, and Woodward–Hoffmann rules.^1−3^ Much of this chemistry is also qualitatively explained by the Hückel model.^4^ Polyenes are also prototypical examples for the use of the particle-in-a-box model where the assumption is that of perfect planarity of the molecules.^5^ Under the condition of planarity, a characterizing feature of polyenic systems is the orthogonality of the σ-electron scaffold and the π-electron frameworks. As the planarity is removed, the alternation of electron density between double and single bonds entails markedly different energy barriers for their isomerization.

Polyenes are also at the core of large numbers of applications that range from organic electronics to the fabrication of sensors and light emitting diodes, to name a few examples.^6,7^ Nature has selected them for a variety of functions that include the vision process and antibiotics.^8−10^ In many of these functions, electric fields play a fundamental role in the definition of the environment or in terms of electromagnetic radiation.

As a rule, it is often assumed that the coupling of molecules with external fields is weak and that the nature of the molecular states is not modified by external agents such as electromagnetic radiation. This assumption is challenged under strong coupling.^11−15^ Strong coupling occurs, for instance, between the vacuum electric field modes present inside the nanosized cavity with reflecting windows, hereafter for simplicity called the cavity, and molecular energy levels. The size of the cavity determines the modes and the type of molecular levels that form the hybrid polaritonic states whose wave function contains molecular and field terms.^16^ Polaritonic states, or polaritons, modify the potential energy landscape, which, in turn, affects reaction pathways and alters reaction rates.^11,17−24^

Although the approach is simplistic, two scenarios emerge. On the one hand, interaction of a molecule with a “weak” electromagnetic field can excite it to different potential energy surfaces. On the other hand, the molecule interaction with a strong field can modify its potential energy surface. The effects could be similar, despite their very different origin. Computationally, it is now possible to simulate, both statically and dynamically,^25−28^ proton transfer,^29^ energy transfer,^30^ electron transfer,^31−33^ intermolecular interactions,^34^ relativistic effects,^35^ ionizations,^36^ and, in general, the effect of a nanoplasmonic cavity on reactivity.^37−41^ In this work, we investigate the effect of the presence of a cavity (and the variation of the molecular orientation) on the torsional degree of freedom of the simplest polyene, namely, butadiene.

We employ a quantum electrodynamics approach to study the effects of the cavity on the isomerization of 1,3-butadiene that is the smallest π-conjugated system that can undergo a low energy isomerization process in the ground state.^42^ Initially, the molecular orientation is chosen to align the dipole moment with the cavity mode direction, resulting in a maximal light–matter coupling strength. We then modify the orientation to show that other potential energy curves can be obtained that are lower in energy. The simulations in the cavity environment are compared with results in the vacuum under the effect of a classical electric field.

A molecular system interacting with quantized light can be modeled using the Pauli–Fierz Hamiltonian, which reads^16^
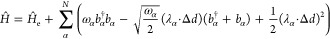
1where *Ĥ*_e_ is the electronic Hamiltonian, the second term describes *N* cavity modes and the matter–cavity mode coupling, *b*_α_^†^/*b*_α_ are the bosonic creation/annihilation operators, λ_α_ is the light–matter coupling strength (also called the coupling factor) between the molecule and the αth mode, Δ*d* = *d* – ⟨*d*⟩ is the dipole fluctuation operator, and ω_α_ is the frequency of the αth mode.

Several methods have been developed to solve the Schrödinger equation associated with the Pauli–Fierz Hamiltonian;^43−50^ in this work we use the quantum electrodynamics Hartree–Fock (QED-HF) method, that is elegantly treated elsewhere.^51,52^

The effect of the cavity can be tuned by changing the coupling strength, that in eq 1 is defined as  where ε_0_ is the vacuum permittivity and *V*_α_^eff^ is the effective quantization volume of the cavity mode.^53^

Throughout this work, we will consider a bimodal cavity where the effective coupling strength is given by .

In order to compare the effects of the quantized field to the electric field in free space, the coupling strength is related to the one-photon field amplitude (*E*) through
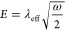
2where we assumed that the field is not position-dependent. In the comparative calculations in free space, eq 2 implies that a single mode represents the classical field. Under this assumption, when computing the amplitude of the associated field, we consider ω = ω_α_.

The calculations in the cavity environment were performed with the *e*^*T*^ program (version 1.9.11);^54^ the calculations with the classical field were carried out with the Gaussian 16 program.^55^ All calculations were performed at the HF/6-31G** level.^56−60^

The isomerization pathway in the cavity is calculated for a number of values of λ_α_ that go from 0.01 to 0.04 in steps of 0.01 (in atomic units). In the calculations in the absence of the cavity, we used the electric field obtained by the fundamental mode employing a value of frequency ω of 0.01 au. This frequency is associated with a wavelength of ∼725 nm. Such a value is far from that of any electronic excitation. The associated values of the electric field range from 0.001 to 0.004 in steps of 0.001 (in atomic units), that is, from 0.514 to 2.057 V/nm.

The conformational isomerization of 1,3-butadiene occurs via a rotation about the central C–C bond. Starting from the s-trans conformer, where conventionally the torsional angle is set to 0°, the rotation leads to a transition state with the largest energy barrier, or TS1, and then to the gauche conformer. The interconversion between the two gauche conformers occurs via a lower energy transition state, or TS2, which is the planar s-cis conformer.^42^

The effect of an electric field also depends on its direction. At equilibrium, the molecular dipole moment orients along it. However, several techniques exist to control molecular orientation,^61−63^ and a change in molecular orientation can enhance/diminish the effect of the coupling and affect a reaction path.^16,37,38,43,53^

Figure 1 shows a summary of the potential energy curves. The torsional potential energy curves are calculated with the field oriented along the axes *x* in Figure 1a, *y* in Figure 1b, and *z* in Figure 1c. In Figure 1d, the field is oriented along the molecular dipole moment. In Figure 1e the initial point is taken with the field aligned along the eigenvector of the polarizability tensor associated with the lowest eigenvalue.

**Figure 1:**
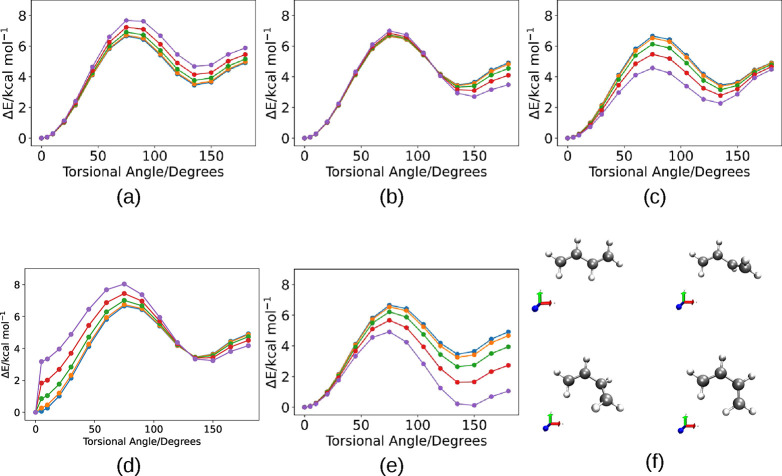
Potential energy curves of the torsion of the central bond of butadiene. The cavity mode/electric field is oriented along (a) the *x*-direction, (b) the *y*-direction, (c) the *z*-direction, or (d) the direction of the molecular dipole moment vector, or (e) with the direction of the molecular dipole moment vector at the origin, the field is aligned along the eigenvector of the polarizability tensor associated with the lowest eigenvalue. Blue: λ_α_ = 0.0/*E* = 0.0 (free space); orange: λ_α_ = 0.01 au = 8.73 × 10^18^ F^–1/2^ m^–1^/*E* = 0.001 au = 0.514 V/nm; green: λ_α_ = 0.02 au = 1.77 × 10^19^ F^–1/2^ m^–1^/*E* = 0.002 au = 1.028 V/nm; red: λ_α_ = 0.03 au = 2.62 × 10^19^ F^–1/2^ m^–1^/*E* = 0.003 au = 1.542 V/nm; purple: λ_α_ = 0.04 au = 3.49 × 10^19^ F^–1/2^ m^–1^/*E* = 0.004 au = 2.057 V/nm. (f) Selected conformations of 1,3-butadiene together with the axis orientation: red: *x*; green: *y*; blue: *z*.

At the relatively low values of coupling 0.01 ⩽ λ_α_ ⩽ 0.04, the energy barriers are increased or lowered as a function of the field/molecule relative orientation. No major disruption of the potential energy curves occurs. As a rule, the field–molecule interaction is maximal when the dipole moment and the field are parallel to one another. However, analysis of Figure 1 shows that the minimum energy path does not depend on this simple rule. It is interesting to notice that in some cases there is a sudden increase of the potential energy as the cavity field is switched on close to the s-trans conformation. We notice that this conformation has a null dipole moment that is at odds with all other geometrical structures along the isomerization pathway. It would be advisable to verify the presence of this effect using a more refined quantum chemical method; however, as it stands, the polaritonic states of molecules with a null dipole moment can behave as on–off switches.

An increase in the coupling with the cavity field oriented along the long molecular axis, Figure 1a, increases both barriers. Alternatively, the coupling with cavity field oriented along the short molecular axis affects only the lower TS2 barrier. In practice, the region around the gauche conformation is flattened and TS2 tends to disappear. The coupling with the cavity field oriented perpendicularly to the molecular plane decreases the energy barrier of TS1. The cavity field oriented along the molecular dipole moment increases the energy of TS1 and flattens TS2.

It is important to mention that all calculations were also performed with field values corresponding to that of the fundamental cavity mode but in the absence of the cavity. No variation of the potential energy curves was observed. This is an important result. It shows that the formation of the polariton is required to affect the energy landscape. Higher values of the electric field are required to modify chemistry.^64−70^ The issue is important to report but will not be further discussed.

Figure 2 shows the electron density variation that occurs upon switching on the coupling. For the sake of clarity, the value of λ_α_ was set to 0.04. In the top panels, for the field oriented along the molecular axis, the binding density increases for both the s-trans and the s-cis conformers. On the contrary, for the field oriented perpendicularly to the molecular axis, the binding density decreases for both conformations. These trends are also present for TS1. Inspection showed that the variation of the binding density is not related to modification of the π orbitals; rather, it is the σ framework that is affected. In this respect, the cavity plays a role substantially different from that of electron excitation. In the absence of the cavity, a torsional energy barrier arises during the rotation by making the two π-bonds orthogonal and is therefore due to confinement. In the presence of coupling, instead, strengthening or weakening of binding arises because of mixing of the σ–π systems.

**Figure 2:**
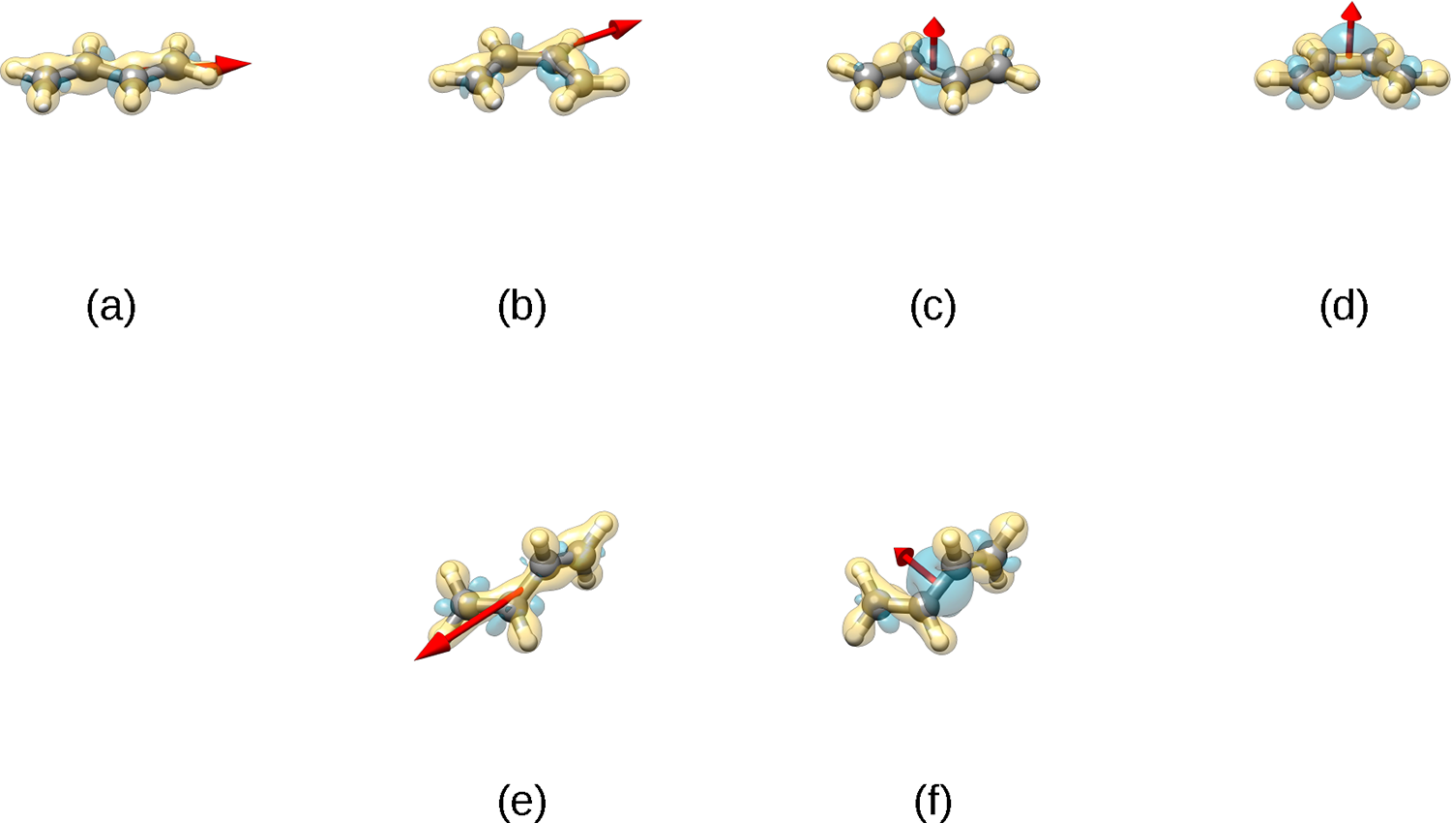
Electron density variation upon switching on the coupling λ_α_ = 0.04 au = 3.49 × 10^19^ F^–1/2^ m^–1^ for the planar s-trans and s-cis conformations (TS2) and TS1. Yellow indicates regions of higher binding; blue indicates lower binding. The cavity modes are oriented along the arrow: (a) as in Figure 1a; (b) as in Figure 1a; (c) as in Figure 1c; (d) as in Figure 1c; (e) as in Figure 1a; (f) as in Figure 1c.

Because of their apparent simplicity, polyenes are ideal species to investigate fundamental effects. Their photophysics has received a great deal of attention, while the effect of electric fields on their dynamics is only emerging.^1^

In this study, we investigated the potential energy curves of the isomerization of 1,3-butadiene. The field of a plasmonic cavity alters the curve through a mechanism and at values that have no classical counterpart and couple σ–π frameworks.

## Supporting Information Available

Transparent Peer Review report available (PDF)
