# Supplementary material for: Polaritonic Chemistry: Hindering and Easing Ground State Polyenic Isomerization via Breakdown of σ–π Separation
Source: J Phys Chem Lett. 2023 Oct 5;14(40):9145–9. doi: 10.1021/acs.jpclett.3c02081 (PMC10577679; doi:10.1021/acs.jpclett.3c02081)
Supplement: Supplementary file 1 — jz3c02081_si_001.pdf [file jz3c02081_si_001.pdf]

jz-2023-02081u.R1

Name: Peer Review Information for "Polaritonic Chemistry: Hindering and Easing Ground State Polyenic-isomerization via Breakdown of σ-π Separation"

First Round of Reviewer Comments

Reviewer: 1

Comments to the Author

This work provides a nice and convincing demonstration of how quantum vacuum fluctuations can indeed modify the chemical properties of molecular systems and drive reactions that otherwise are not possible in the gas phase. The present work shown quantum electrodynamical calculations of such a effect to the polyenic-isomerization of butadiene. The work is well written and provides clear evidences for cavity control. As such I consider the paperan excellent contribution to The Journal of Physical Chemistry Letters.

Author's Response to Peer Review Comments:

Your re: jz-2023-02081u

Dear Prof. Dr.,

I would like to thank you for your email of Aug 16 and for considering our work suitable for publication in the Journal of Physical Chemistry Letters.

Please find enclosed the revised version of the manuscript where we have addressed all the editorial concerns.

Sincerely,

Prof. Francesco Zerbetto
